# Non-*Candida albicans Candida* species: virulence factors and species identification in India

**DOI:** 10.18502/cmm.7.2.7032

**Published:** 2021-06

**Authors:** Dharmendra Prasad Singh, Rajesh Kumar Verma, Swati Sarswat, Satender Saraswat

**Affiliations:** 1 Department of Microbiology, Uttar Pradesh University of Medical Sciences, Saifai, India; 2 Department of Community Medicine, Gajra Raja Medical College, Gwalior, India

**Keywords:** ABEC, NCAC, Non-*Candida albicans Candida* species, VITEK-2® Compact

## Abstract

**Background and Purpose:**

The predominant cause of candidiasis was Candida albicans which has recently changed to non-*Candida albicans Candida* (NCAC) (i.e., *Candida* spp. other than the *C. albicans*). The NCAC spp., earlier considered non-pathogenic or minimally virulent, are now considered a primary cause of morbidity and mortality in immunocompromised individuals. Given the NCAC spp.has become more common in clinical cases, this study aimed to determine the prevalence of NCAC spp. in different clinical specimens and assess a few of their virulence factors.

**Materials and Methods:**

Routine samples for bacterial culture and sensitivity that showed colony characteristics, like *Candida* on Blood Agar and microscopic features resembling *Candida* spp., were processed further. *Candida* isolates underwent tests for chlamydospore formation and biochemical tests, including sugar fermentation and sugar assimilation tests. These were grown at 42oC, and their colony color was identified using HiCrome^TM^ Candida Differential Agar (HiMedia Laboratories Pvt. Ltd., Mumbai, India), Hi*Candida*^TM^ Identification Kit (HiMedia Laboratories Pvt. Ltd., Mumbai, India), and VITEK-2® Compact (Biomérieux, France). Virulence factors, such as adherence to buccal epithelial cells (ABEC), biofilm formation, hemolytic activity, and production of coagulase enzyme were also tested.

**Results:**

Mean age of the patients was 38.46 years with a male-female ratio of 1.36:1. In total, 137 *Candida* isolates were recovered; 45.3%, 19.7%, and 13.9% of the isolates were isolated from urine, vaginal swabs, and oropharyngeal swabs, respectively. Moreover, 55 (40.1%) isolates were those of *C. albicans* and 82 (59.9%) isolates belonged to NCAC spp., with *C. tropicalis* (23.4%) contributing highest among NCAC species. Furthermore, *C. albicans* (3; 50%) was the most common spp. in cases of candidemia. Haemolysin production (85.5%) and ABEC (78.2%) were the major virulence factors in *C. albicans*. *C. tropicalis* (59.4%) and *C. dubliniensis* (50%) showed maximum ABEC. Biofilm forming capacity was higher in *C. tropicalis* (78.1%) than *C. albicans* (67%).

**Conclusion:**

Results of this study suggest varied prevalence and virulence based on geographical locations, even within a subcontinent. It clearly indicates the emergence of the NCAC spp. and their predominance in different body fluids. Identification of *Candida* to the spp. level should become a routine in all laboratories.

## Introduction

The incidence of fungal infections is increasing and is directly related to the rising population of immunocompromised individuals which has resulted from altered medical practices, like the use of intensive chemotherapy and immunosuppressive medications [ 1 ]. The most commonly encountered fungi include the *Candida* spp., [ 2 ] which can initiate infections in immunocompetent and immunocompromised individuals, but the incidence is higher in the latter [ 3 ]. The predominant cause of candidiasis was *Candida albicans* which has shifted towards non-*Candida albicans Candida* spp. (NCAC) [ 2 ] (*Candida* spp. other than the *Candida albicans*). The NCAC spp., earlier considered non-pathogenic or minimally virulent, are now considered a primary cause of morbidity and mortality in immunocompromised individuals [ 4 ].

Although *Candida* spp. are commensals colonizing the mucosal surfaces asymptomatically, they can cause significant disability and fatal infections [ 5 ]. They cause several clinical diseases ranging from mucocutaneous infections to life-threatening states like candidemia [ 6 ]. The *C. albicans* causes both mucocutaneous and disseminated infections, but infections due to NCAC spp. are rising now [ 6 ]. Various factors, like severe immunocompromised states, use of broad-spectrum antibiotics, prematurity, and empirical use of antimycotics, prolonged hospitalization, presence of intravenous line and Foley's catheter, and immunosuppressive regimen, are associated with augmenting candidiasis associated with NCAC spp. 

Despite the fact that clinical manifestations of infections associated with the different members of NCAC spp. are usually indistinguishable, several NCAC spp. either acquire resistance over time or have ingrained resistance against routine antifungals and sometimes both [ 7 ]. Several virulence factors facilitate the transition of *Candida* spp. from commensal to a potent pathogen, including adherence to host tissues and medical devices, formation of biofilm, secretion of extracellular hydrolytic enzymes (e.g., proteases, lipases, phospholipases, esterases, and phosphatases), toxins, complement receptors, and phenotypic switching [ 6 ]. 

Extensive research has been carried out to determine the pathogenicity of *C. albicans*, but this is not the case with NCAC spp. [ 8 ]. Today, the nature and expression of infectious diseases have undergone a change [ 2 ]. Microorganisms with minimal or no pathogenic role in causing infections have emerged as potent pathogens related to morbidity and mortality throughout the world [ 2 ]. The incidence rate of *C. glabrata* is high in the USA, whereas Latin America and Asia-Pacific countries have a higher incidence of *C. tropicalis* and *C. parapsilosis* [ 9 ]. 

Furthermore, *C. glabrata* and *C. parapsilosis* are among the most common pathogens in Europe [ 9 ]. *C. parapsilosis* has a significant incidence in Mediterranean countries [ 9 ]. In recent years, *Candida* spp. have emerged as a potent human pathogen. Since the NCAC spp. are becoming common in clinical cases, this study was conducted at a tertiary care hospital to determine the prevalence of NCAC spp. in different clinical specimens and to assess a few of their virulence factors.

## Materials and Methods

This cross-sectional study was conducted in the Department of Microbiology, Uttar Pradesh University of Medical Sciences, Saifai, India from January 2020 to December 2020. Prior informed consent and demographic characteristics of the subjects were collected. The study was approved by the Institutional Ethics Committee (Clearance Code: 102/2019-20).

### Samples

This study was performed on routine samples received in the Microbiology laboratory for bacterial culture and sensitivity. The inclusion criteria were possession of colony characteristics similar to that of *Candida* on blood agar after incubation at 37oC, and microscopic features resembling *Candida* spp. after Gram staining. The specimens, like urine, oropharyngeal swab, vaginal swab, blood, ascitic fluid, and pleural fluid were tested. Urine samples were also inoculated on Cysteine-Lactose-Electrolyte-Deficient Agar for semi-quantitative analysis to determine the presence of a significant colony count [ 10 ] of *Candida* spp. Data regarding clinical features supporting the diagnosis of urinary tract infection and demographic characteristics were also collected.

### Isolation

Colonies from Blood Agar were inoculated onto plain Sabouraud’s Dextrose Agar (SDA) and incubated at 37 °C for 24-48 h [ 11 ] or until the creamy white, pasty, and smooth growth was visible, whichever was earlier. Pasty, opaque, smooth, slightly domed or flat, and pale- colored red (i.e., white, off-white, or beige) colonies with a sweet smell evocative of ripe apples were suspected as *Candida* colonies [ 12 ].

### Identification

The identification was done as per the instructions given by Deorukhkar et al., [ 2 ] with some modifications. *Candida* isolates producing germ-tubes within 2 h of incubation underwent chlamydospore formation test as well as biochemical tests, including sugar fermentation and sugar assimilation tests, and were grown at 42 oC to distinguish *C. albicans* from *C. dubliniensis*. Germ-tube negative isolates were classified based on sugar assimilation and colony color on HiCrome^TM^ Candida Differential Agar (HiMedia, Mumbai, India). Identification was supplemented by Hi*Candida*^TM^ Identification Kit (HiMedia Laboratories Pvt. Ltd., Mumbai, India) and VITEK-2® Compact (Biomérieux, France) Systems.

### Virulence Factors

Recovered isolates were screened for some of the virulence factors, like adherence to buccal epithelial cells (ABEC), biofilm formation, hemolytic activity, and production of coagulase enzyme.

### Adherence to buccal epithelial cells

Methods employed by Kimura and Pearsall [ 13 ] were used with minor modifications. Fresh buccal epithelial cells (BECs) were collected by gently rubbing the cotton swabs on the cheek mucosa of healthy donors (i.e., having no clinical features of oropharyngeal candidiasis [OPC] or other oral lesions, and not taking any antibiotics for over a month) after informed consent was obtained from them. 

The BECs were washed by phosphate-buffered saline (PBS) (HiMedia Laboratories Pvt. Ltd., Mumbai, India). Afterward, 1 mL BEC (1×10^5^ cells/mL) and 1 mL yeast suspension (1×10^7^cells/mL) were mixed in equal proportion and incubated at 37 oC for 2 h in a shaking water-bath at 40 rpm. This mixture was filtered through a 20 𝜇m filter (Hyclone Life Sciences Solutions India Pvt. Ltd, Bangalore, India) to remove free yeast cells. The BECs left on the filter were washed with 5 mL PBS and finally suspended in 5 mL PBS.

A drop of this suspension was put on a clean glass slide, and the smear was fixed by methanol, air-dried, and Gram’s stained. Adherence was determined microscopically by counting the number of yeast cells adhering to every 100 BECs and determining their mean value. The control strain used was *C. albicans* ATCC90028.

### Biofilm formation

It was tested by the tube method according to Yigit et al.[ 14 ] with few modifications. *Candida* colonies were inoculated in normal saline and incubated for 24 h at 35 oC. Afterward, 1.5 ml of this suspension was transferred to screw-capped conical polystyrene tube containing 5 mL of Sabouraud’s dextrose broth supplemented with glucose (final concentration: 8%). 

The tubes were incubated at 37 oC for 24 h without agitation , and after the incubation, the broth from the tube was gently aspirated using a Pasteur pipette. The tube was washed thrice with PBS (pH 7.2) and stained with 1% Safranine. The stain was decanted after 15 min and the tube was rinsed with PBS to remove the excess stain. The visible adherent film on the wall and the bottom of the tube indicated biofilm formation. Formation at the liquid interface was considered insignificant. It should be mentioned that *C. albicans* ATCC90028 and *C. albicans* ATCC10231 were used as controls, respectively.

### Hemolytic activity

The hemolytic activity was tested as per the instructions given by Luo et al., [ 15 ]. For this purpose, 10 𝜇L of standard inoculum (10^8^*Candida* cells/ml) was inoculated on sheep blood SDA plates and incubated at 37 oC in 5% CO_2_ for 48 h. The presence of a distinct translucent halo around the inoculum site indicated hemolysis on visualization with transmitted light. The *C. albicans* ATCC90028 and *C. parapsilosis* ATCC22019 were used as positive and negative controls, respectively. In addition, *Streptococcus pyogenes* (Lancefield group A) and Streptococcus sanguis were used as positive controls for beta and alpha hemolysis, respectively.

Production of Coagulase enzyme

The method described by Rodrigues et al., [ 16 ] was employed for the production of the coagulase enzyme. In this regard, 100 μl of an overnight culture of *Candida* isolate was aseptically inoculated into a test tube containing 500 μl of EDTA-rabbit plasma. The tube was incubated at 37 oC in a water bath and observed for clot formation at 1 h, 2 h, and 4 h [ 17 ]. The visible clot that could not be resuspended by gentle shaking indicated a positive test. If no clot was formed, the tube was re-incubated and re-examined at 24 h. The *Staphylococcus aureus* ATCC25923 and *Staphylococcus epidermidis* ATCC14990 were positive and negative controls, respectively.

## Results

In this study, the mean age of the patients was 38.46 (range: 7-84 years) with a male-female ratio of 1.36:1. In total, 137 *Candida* isolates were recovered from various clinical specimens and most (45.3%) of them were isolated from urine, followed by vaginal swabs (19.7%) and oropharyngeal swabs (13.9%). Moreover, 55 (40.1%) isolates were identified as *C. albicans* while 82 (59.9%) of them belonged to NCAC spp., among which, *C. tropicalis* (23.4%) was the most common spp. followed by *C. glabrata* (14.6%) and *C. krusei* (11.7%). 

It should be mentioned that *C. albicans* (3; 50%) was the most common spp. in cases with candidemia followed by *C. glabrata* (2; 33.3%) and *C. tropicalis* (1; 16.7%). In the Ascitic fluid, six isolates of *C. albicans* (40%) and nine isolates of NCAC spp. (60%) were recovered, the most common of which was *C. krusei* (44.4%), followed by *C. tropicalis* (33.3%) (Table 1).

**Table 1:** Clinical sample-wise distribution of Candida isolates

Clinical samples	Urine	Oropharyngeal Swab	Vaginal Swab	Blood	Ascitic Fluid	Pleural fluid	Total isolates
Species							
*Candida albicans*	23	9	11	3	6	3	55 (40.1%)
*Candida tropicalis*	18	3	6	1	3	1	32 (23.4%)
*Candida glabrata*	11	2	3	2	1	1	20 (14.6%)
*Candida krusei*	6	2	2	0	4	2	16 (11.7%)
*Candida kefyr*	3	1	1	0	0	1	6 (4.3%)
*Candida parapsilosis*	1	0	2	0	1	0	4 (2.9%)
*Candida guillermondii*	0	1	1	0	0	0	2 (1.5%)
*Candida dubliniensis*	0	1	1	0	0	0	2 (1.5%)
Total specimen	62 (45.3%)	19 (13.9%)	27 (19.7%)	6 (4.4%)	15 (10.9%)	8 (5.8%)	137 (100%)

In total, 118 patients (86.13%) were rural residents, 29 patients (21.16%) had a previous history of recurrent systemic infections (≥3 episodes) in the past 1 year, 69 patients (50.36%) received antibiotics in the last 3 months, 23 patients (16.78%) reported consumption of antifungals (for superficial mycosis), 17 patients (12.40%) reported corticosteroid use, 39 patients (28.46 %) had diabetes mellitus, 14 patients (10.21%) were bed-ridden and on intensive management.

Haemolysin production (85.5%) followed by ABEC (78.2%) were the major virulence factors in the *C. albicans*. Among NCAC spp., *C. tropicalis* (59.4%) and *C. dubliniensis* (50%) showed maximum ABEC. Biofilm forming capacity was higher in *C. tropicalis* (78.1%) than in *C. albicans* (67%) while it was absent in *C. guillermondii* and *C. dubliniensis*. It should be noted that *C. guillermondii* showed no ABEC either. Coagulase production was absent in *C. kefyr*, *C. parapsilosis*, and *C. dubliniensis* (Table 2).

**Table 2:** Virulence factors exhibited by Candida isolates

Candida species	Adhesion to Buccal Epithelial Cells (%)	Biofilm formation (%)	Coagulase production (%)	Haemolysin production (%)
*Candida albicans*	43 (78.2)	37 (67)	32 (58.2)	47 (85.5)
*Candida tropicalis*	19 (59.4)	25 (78.1)	18 (56.3)	19 (59.4)
*Candida glabrata*	12 (30)	13 (65)	9 (45)	14 (70)
*Candida krusei*	5 (31.3)	4 (25)	3 (18.8)	5 (31.3)
*Candida kefyr*	1 (17)	1 (17)	0	1 (17)
*Candida parapsilosis*	1 (25)	1 (25)	0	1 (25)
*Candida guillermondii*	0	0	1 (50)	1 (50)
*Candida dubliniensis*	1 (50)	0	0	1 (50)

## Discussion

In this study, urine was the primary sample from which Candida isolates were obtained. It was found that 23 (37%) of them were *C. albicans*, while 39 (63%) were NCAC spp. Based on the findings of the studies conducted by Deorukhkar et al., [ 2 ], Alvarez-Lerma et al., [ 18 ], and Kauffmann [ 19 ], more than 50% of urinary *Candida* isolates belonged to NCAC spp. Similar results were obtained in the present study as well. In another study performed by Toner, L et al. [ 20 ], it was revealed that the most common NCAC spp. in cases of candiduria were *C. glabrata*, *C. tropicalis*, and *C. parapsilosis*. In the present study, *C. tropicalis* was the most prevalent spp. followed by *C. glabrata* and *C. krusei*.

According to the findings of a study carried out by Gharghani M et al., [ 21 ], host factors (i.e., genitourinary abnormality, diabetes mellitus, and immunodeficiency) and invasive therapy (i.e., indwelling urinary catheters, widespread systemic antibiotic, surgery, and chemotherapy) play a role in the rising prevalence of candiduria. In another study performed by Paul N et al. [ 22 ], prior use of antimicrobial agents and elevated plasma glucose were stated as the risk factors for candiduria. Results of the present study were in line with those of both of the above-mentioned studies and included urinary catheterization, extremes of ages, diabetes mellitus, pregnancy, patients on antibiotics, and intensive management as the risk factors.

Common features of mucosal candidiasis include OPC and vulvovaginal candidiasis (VVC). The VVC is usually diagnosed by clinical examination with minimal or no laboratory support. Studies performed by Deorukhkar et al. [ 2 ] and Mohanty et al. [ 23 ] identified the majority of the isolates as *C. glabrata* and *C. tropicalis* in the NCAC spp. group. Similar results were observed in the present study which constituted 59.3% of the NCAC spp., the most common of them being *C. tropicalis* (37.5%) and *C. glabrata* (18.8%). 

The most common opportunistic mycoses in immunocompromised individuals are the OPC. Results of a study [ 24 ] indicated the highest incidence of C. albicans and C. guillermondii in OPC. In the present study, most of the NCAC spp. (10; 52.6%) isolates were C. tropicalis. It should be mentioned that among the cases of OPC, 11 cases (57.9%) were HIV positive.

Few studies [ 2 , 3 ] have reported an increasing prevalence of *C. glabrata* in disseminated infections, while one study [ 25 ] stated a higher incidence rate of *C. albicans* in candidemia. In the present study, *C. glabrata* was the most common spp. in cases of candidemia. The risk factors causing *C. glabrata* bloodstream infection are similar to other spp. of *Candida*, but the former spp. has a higher mortality rate [ 3 ]. The transition of commensals into potential pathogens is determined by several host factors and the virulence of the pathogen [ 6 ]. Identification of these virulence factors and determination of their effects on the host is essential. 

The ABEC of *Candida* spp. to the epithelial cells of hosts is crucial in the pathogenesis of its infection. Adhesion to host cells, host cell proteins, or microbial competitors prevents and/or reduces the extent of clearance by the defense mechanisms of the host [ 26 ]. Some studies [ 2 , 27 ] have suggested the highest ABEC in *C. albicans*, which is in line with the results of this study. Among NCAC spp., *C. tropicalis* followed by *C. glabrata* and *C. dubliniensis* demonstrated high adherence to the BEC of the host.

An increase in hospital admission, medical advancement, use of antimicrobials, along with the evolved adaptation of microorganisms to the healthcare environment, have contributed to increased healthcare-associated infections (HCAIs). *Candida* spp. have a miraculous adaptive capacity regarding a variety of habitats, including various medical devices, causing the emergence of *Candida* spp. as the main spp. isolated from HCAIs. 

They can form biofilm on most medical devices [ 28 ] and are the surface communities of microorganisms constituting the extracellular matrix [ 29 ]. The increasing popularity of venous catheters is one of the foremost causes of increased incidence of NCAC spp. and declining incidence of *C. albicans* [ 30 ]. In this study, biofilm-formation was mostly observed in *C. tropicalis* (78.1%), *C. albicans* (67%), and *C. glabrata* (65%). Biofilm increases the ability of *Candida* to withstand host defense and also develop resistance against antifungals [ 6 ]. Strains of Candida with biofilm-forming ability are associated with higher morbidity and mortality. The formation of mature biofilm and production of the extracellular matrix depends on the spp., strain, and environmental conditions [ 29 ].

The production of hydrolytic enzymes is crucial in the pathogenesis of *Candida* and depends on the pathogen spp., source of infection, and site of infection [ 31 ]. The coagulase enzyme binds with the plasma fibrinogen resulting in activation of a reaction cascade, thereby initiating the plasma coagulation [ 14 ]. Deorukhkar ,.et al. [ 2 ] and Yigit et al., [ 14 ] stated the highest coagulase activity in *C. albicans* (64.7%) and *C. kefyr* (42.8%), while it was highest in *C. albicans* (58.2%) and *C. tropicalis* (56.3%) in the present study.

Haemolysin utilizes iron contained in the hemoglobin and activates complement to opsonize the surface of red blood cells (RBCs) [ 31 ]. This destroys host RBCs and facilitates hyphal invasion in systemic candidiasis [ 6 , 14 ]. Hemolysin is important for pathogenicity as it imparts the survival capacity to the Candida. Based on the findings of a previous study [ 32 ], the highest hemolytic activity was observed in *C. glabrata* (72.7%), *C. tropicalis* (72.7%), and *C. albicans* (66.6%), while in the present study, it was the highest in *C. albicans* (85.5%), *C. glabrata* (70%) and *C. tropicalis* (59.4%). As it was a laboratory-based study, the actual prevalence of *Candida* infection in the community could not be determined, and it was further supplemented by the COVID-19 pandemic and lock downs throughout India. Given the above-mentioned factors, much of the sampling was reduced and further prospective work-up, including antifungal susceptibility, could not be performed.

## Conclusion

The mean age of patients with candidiasis was 38.46 (range 7-84) years with a male-female ratio of 1.36:1. In this study, the highest predominance of *Candida* was detected in urine, followed by the vaginal swab. Moreover, it was found that *C. tropicalis*, *C. glabrata*, and *C. krusei* had the highest incidence rate among the NCAC spp. Given the virulence factors, ABEC was highest in *C. albicans*, *C. tropicalis*, and *C. glabrata*; biofilm-formation was highest in *C. tropicalis*, *C. albicans*, and *C. glabrata*. In addition, coagulase production was highest in *C. albicans* and *C. tropicalis*; and hemolysis was highest in *C. albicans*, *C. glabrata*, and *C. tropicalis* in this study. 

The findings suggest varied prevalence and virulence based on geographical locations, even within a subcontinent. It clearly demarcates the emergence of NCAC spp. and their predominance in different body fluids. Risk factors of candidiasis need to be kept in mind for a clinician as well as the laboratory personnel. Identification of *Candida* to the spp. level should become a routine in all the laboratories using VITEK-2® Compact (Biomérieux, France), molecular techniques, or other advanced methods. Furthermore, a study with a greater number of samples with follow-up providing an outcome would aid in the better realization of prognosis and response.

## Acknowledgement

The authors acknowledge the efforts of all the members of the Microbiology Department, especially Dr. Vikas Yadav, as well as the study participants.

## Authors’ contribution

Concept and design: D.P.S; data collection and processing : S. S.; analysis and interpretation : R.K.V.; writing manuscript: S. S. and S. S.; critical review : D.P.S. and R.K.V.

## Conflict of Interest

The authors have no conflict of interest.

## Financial disclosure

The authors declare that they have not received any financial support for this study.
